# Diagnostic dilemma: Differentiating coexisting systemic lupus erythematosus and Sjögren’s syndrome mimicking multiple myeloma in a patient with hypergammaglobulinemia presenting to primary care – A case report

**DOI:** 10.5339/qmj.2025.91

**Published:** 2025-09-09

**Authors:** Zhila Mohammed, Zozik Fattah, Anas Kalfah, Hassan Ibrahim, Alan Saeed

**Affiliations:** 1 Master’s in Assisted Reproductive Technology (University of Nottingham, UK) College of Medicine, Qatar University, Doha, Qatar; 2 Consultant Rheumatologist Mediclinic City Hospital, Dubai, UAE; 3 Resident Doctor, Medical Education Department Hamad Medical Corporation, Doha, Qatar; 4 Hamad Medical Corporation, Doha, Qatar; 5 Consultant Family Physician [MBChB, FRCP (UK), MRCGP (UK), MRCP (Diabetes & Endocrinology), DFSRH] Primary Health Care Corporation, Doha, Qatar *Email: alsaeed@phcc.gov.qa

**Keywords:** Systemic lupus erythematosus, Sjögren’s syndrome, hypergammaglobulinemia, diagnostic challenges, autoimmune diseases

## Abstract

**Introduction:**

Systemic lupus erythematosus (SLE) and Sjögren’s syndrome (SS) are chronic autoimmune diseases that can coexist, complicating the diagnostic process due to overlapping clinical and serological features. Hypergammaglobulinemia, often associated with these conditions, can mimic hematological malignancies, posing a significant diagnostic challenge.

**Case Presentation:**

We report a case of a 57-year-old woman presenting with progressive fatigue, dry cough, and vasculitic rashes for several weeks. Initial laboratory results revealed pancytopenia, hypergammaglobulinemia, and elevated inflammatory markers (erythrocyte sedimentation rate (ESR) >100 mm/hr, C-reactive protein (CRP) 137.7 mg/L), raising suspicion for multiple myeloma. However, protein electrophoresis (which showed polyclonal rather than monoclonal globulinemia) and bone marrow biopsy excluded malignancy. Further autoimmune screening confirmed coexisting SLE and SS, with positive ANA (≥1:1280), anti-dsDNA, anti-Ro, and anti-La antibodies. Polyclonal hypergammaglobulinemia was attributed to these autoimmune conditions. The patient responded well to azathioprine and hydroxychloroquine, with significant clinical and biochemical improvement.

**Discussion:**

This case highlights the importance of distinguishing polyclonal hypergammaglobulinemia due to autoimmune diseases from monoclonal gammopathies such as multiple myeloma. Early autoimmune screening and multidisciplinary collaboration were essential in achieving an accurate diagnosis and appropriate management.

**Conclusion:**

Polyclonal hypergammaglobulinemia in the presence of elevated inflammatory markers warrants a thorough differential diagnosis to distinguish autoimmune conditions from hematological malignancies. This case underscores the critical role of comprehensive autoimmune screening in guiding timely and accurate diagnosis.

## 1. INTRODUCTION

Systemic lupus erythematosus (SLE) and Sjögren’s syndrome (SS) are chronic autoimmune disorders that share clinical and serological features, often complicating the diagnostic process. SLE is a multisystem inflammatory disease that predominantly affects women of childbearing age, with a female-to-male ratio of approximately 9:1.^1,2^ Its pathogenesis involves a combination of genetic predisposition, hormonal factors, and environmental triggers, resulting in B-cell hyperactivity and autoantibody production.^3^ Common clinical manifestations include fatigue, joint pain, rash, nephritis, and neurological symptoms. Disease activity is typically monitored through markers such as anti-dsDNA antibodies, complement levels, and inflammatory markers such as erythrocyte sedimentation rate (ESR) and C-reactive protein (CRP).^4^

B cells play a crucial role in the adaptive immune response by producing FLCs, which serve as biomarkers for disease activity in various inflammatory and autoimmune conditions.^5^ In SLE, elevated levels of polyclonal FLCs are mainly due to the enhanced B-cell response during inflammation.^6^ The balance between B-cell synthesis and renal clearance determines the circulating levels of FLCs, which are swiftly removed from the circulation by the kidneys within 2–6 hours.^7^ During active disease periods, urinary FLC levels increase significantly, reflecting ongoing inflammation and B-cell activity.^7^ Serum concentrations of FLCs also rise in SLE patients during active disease phases, correlating with inflammation markers such as complement consumption and high CRP levels.^7^

SS is characterized by lymphocytic infiltration of exocrine glands, leading to sicca symptoms, including dry eyes and mouth. It may occur as a primary condition or secondary to other autoimmune diseases, such as SLE or rheumatoid arthritis. The hallmark antibodies of SS, anti-Ro (SSA) and anti-La (SSB), are present in 40%–80% of cases.^8^ SS also presents with systemic features, such as arthritis, vasculitis, and a predisposition to low-grade lymphomas, including mucosa-associated lymphoid tissue (MALT) lymphoma, with a lifetime risk of 5%–7.5%.^2^

Polyclonal hypergammaglobulinemia is a distinctive feature of SS and serves as a predictive marker for the risk of developing lymphoma, particularly low-grade non-Hodgkin lymphoma and mucosa-associated lymphoid tissue lymphoma, with an overall risk of 5%–7.5%.^2^ SS can also cause non-inflammatory joint pain in about 75% of patients and inflammatory arthritis in approximately 10%. Other manifestations include dry skin (xeroderma), annular erythema, swollen salivary glands, dry cough, recurrent bronchitis, and pneumonias.^9^ Oral complications such as dental cavities and oral thrush are common due to reduced mucosal immunity and dryness.^10^ Visual complications, renal involvement (chronic tubulointerstitial nephritis), and neurological symptoms (peripheral neuropathy) are also associated with SS.^2,11^

The coexistence of both SLE and SS is observed in 9–33% of cases, attributed to shared genetic factors, immune dysregulation, and environmental influnces.^12^ Specifically, polymorphisims and the HLA-DR locus, particularly the presence of HLA-DR3, are associated with both conditions. Immune dysregulation in both conditions involves the critical role of B cells in disease progression through the production of autoantibodies such as anti-SSA/Ro and anti-SSB/La, which are found in both SLE and SS.^12^ Both SLE and SS are associated with environmental triggers, including viral infections (e.g., Epstein-Barr virus), hormonal influences, and smoking.^13,14^ Infections can activate immune pathways that predispose individuals to these conditions, whereas hormonal factors contribute to their higher prevalence in women with SLE peaking during childbearing age and SS often emerging post menopause.^14^

Patients with hypergammaglobulinemia and pancytopenia often require a meticulous diagnostic workup to distinguish between autoimmune diseases and hematologic malignancies. Monoclonal gammopathies, such as multiple myeloma and monoclonal gammopathy of undetermined significance (MGUS), are commonly considered due to their potential for significant morbidity. However, polyclonal hypergammaglobulinemia, as seen in autoimmune diseases, can mimic the laboratory and clinical features of these malignancies.^15^

Differentiating SLE and SS from multiple myeloma (MM) can be challenging due to overlapping clinical features and lab findings. Although rare, the simultaneous occurrence of MM and SLE has been reported in several case reports.^16^ SLE is associated with a higher risk of malignancies, particularly hematologic cancers such as non-Hodgkin lymphoma, Hodgkin lymphoma, and plasma cell disorders, including multiple myeloma.^16,17^

Both SLE and SS can present with fatigue, anemia, and arthralgia, which are also common in MM. Only a few similar cases reported and described a case where a patient with SLE and SS was initially misdiagnosed as having multiple myeloma due to elevated serum protein levels and abnormal electrophoresis. The polyclonal immunoglobulin elevation seen in SLE and SS was mistaken for the monoclonal protein associated with multiple myeloma. The correct diagnosis of SLE and SS was confirmed only after further tests, including bone marrow biopsy and serological markers.^16^

This case report explores a diagnostic dilemma involving a 57-year-old woman who presented with polyclonal hypergammaglobulinemia, pancytopenia, and elevated inflammatory markers. The initial suspicion of multiple myeloma was later revised to a diagnosis of coexisting SLE and SS. This report highlights the critical role of comprehensive autoimmune screening and collaborative care in achieving an accurate diagnosis and appropriate management.

## 2. CASE PRESENTATION

A 57-year-old woman from the Philippines presented with a two-month history of progressive fatigue, persistent dry cough, and shortness of breath. She also reported vasculitic rashes on both legs (livedo reticularis) for several weeks. Her medical history included rheumatic mitral valve stenosis, for which she had declined surgery. She was not on any regular medications, had no known drug allergies, and had no significant family medical history.

## 3. CLINICAL FINDINGS

The patient appeared pale on examination but was otherwise hemodynamically stable, with no jaundice, cyanosis, or clubbing. Palpation revealed cervical lymphadenopathy, with nodes measuring 1–2 cm that were mobile and soft in consistency. Dermatological examination revealed a livedo reticularis rash on both legs. Cardiac auscultation identified a mid-diastolic murmur radiating to the left axilla, consistent with her known mitral valve stenosis. Respiratory and abdominal examinations were unremarkable, with no organomegaly or tenderness.

## 4. LABORATORY RESULTS

Initial investigations revealed several abnormalities (Table 1):

- Hemoglobin: 9.3 g/dL (reference range: 12.5–18.0 g/dL), consistent with microcytic anemia.
- White blood cell count: 3.3 × 10⁹/L (reference range: 4–11 × 10⁹/L), indicating leukopenia.
- Platelet count: 122,000 K/uL (reference range: 150–400 K/uL), indicating mild thrombocytopenia.
- ESR: 105 mm/hr (reference range: <30 mm/hr) and CRP: 137.7 mg/L (reference range: 0–5 mg/L), both markedly elevated.
- Serum total protein: 107 g/L (reference range: 60–80 g/L), with hypoalbuminemia (32 g/L; reference range: 35–50 g/L), consistent with hyperproteinemia.
- Peripheral smear: showed leukopenia, monocytopenia, thrombocytopenia, and red cell abnormalities, including anisocytes, teardrop cells, schistocytes, and target cells.

## 5. FURTHER DIAGNOSTIC WORKUP

Given the marked hypergammaglobulinemia, pancytopenia, and systemic inflammatory markers, a comprehensive workup was undertaken to exclude hematologic malignancies and autoimmune diseases. The diagnostic approach included serological, protein electrophoretic, genetic, and imaging evaluations (Table 2). Key findings included:

- Protein electrophoresis: Demonstrated a dense polyclonal increase in gamma globulins without a monoclonal band, strongly indicative of a chronic inflammatory process rather than clonal plasma cell pathology, effectively ruling out multiple myeloma.
- Free Light Chain Assay: Results showed Kappa: 43 and Lambda: 30 with a normal Kappa/Lambda ratio, further supporting a polyclonal process and reducing suspicion of monoclonal gammopathies.
- Autoimmune Screening: Strongly positive antinuclear antibody (ANA) (≥1:1280, speckled pattern), anti-dsDNA (1600 IU/mL), anti-Ro, and anti-La antibodies confirmed SLE and SS as the underlying etiology of the patient’s systemic findings.

In addition, genetic testing was conducted to exclude hematologic malignancies such as myeloproliferative neoplasms, given the hypermetabolic activity noted on the fluorodeoxyglucose positron emission tomography/computed tomography scan (FDG PET-CT) and the hematological abnormalities:

- JAK2 V617F Mutation Analysis: No evidence of the V617F missense mutation was identified, effectively ruling out polycythemia vera and other JAK2-related neoplasms.
- CALR Mutation Analysis: No insertions or deletions were detected in exon 9 of the CALR gene, further excluding the possibility of essential thrombocythemia or primary myelofibrosis.

These genetic analyses were performed because the hypermetabolic activity in the cervical lymph nodes, L4 vertebra, and left adrenal gland could have indicated an underlying clonal hematological process despite initial findings from bone marrow aspiration and biopsy. Their absence reinforced the conclusion that the patient’s presentation was due to autoimmune pathology rather than malignancy.

The general immunological result showed increased IgG and IgA immunoglobulin levels, with a normal IgM level, and an increase in C4 complement protein (Table 3).

## 6. IMAGING AND BIOPSY

Advanced imaging and biopsy results (Table 4) further clarified the diagnosis:

- **Echocardiography:** Demonstrated increased pulmonary artery pressure and mild left ventricular dysfunction (LVEF 54%), consistent with rheumatic mitral valve disease.
- **FDG PET-CT:** Showed hypermetabolic activity in cervical lymph nodes, the L4 vertebra, and the left adrenal gland but no high-grade malignancy.
- **Bone marrow biopsy:** Showed trilineage hematopoiesis with mild fibrosis and multiple lymphoid aggregates, excluding multiple myeloma.
- **Fine-needle aspiration of the right submandibular lymph node:** Revealed benign/reactive lymphoid hyperplasia.

## 7. DIAGNOSTIC ASSESSMENT AND INTERPRETATION

In the primary care setting, the initial laboratory investigations included a complete blood count, inflammatory markers (ESR and CRP), metabolic panel, and liver and renal function tests. The results showed anemia, hypercalcemia, and pancytopenia with ESR over 100 and high total protein but low albumin, indicating high circulating globulin proteins. These findings were highly suspicious for multiple myeloma, leading to a two-day urgent hematological cancer referral. Further investigations, including protein electrophoresis, revealed polyclonal hypergammaglobulinemia, reducing the likelihood of multiple myeloma.

In the secondary care hematology clinic, bone marrow aspiration and peripheral blood studies ruled out multiple myeloma. The patient was then referred to rheumatology, where comprehensive rheumatological studies, including autoantibody screening, confirmed SLE and SS. Positive ANA antibodies and anti-dsDNA indicated SLE, whereas positive anti-La and anti-RO antibodies confirmed SS. The polyclonal hypergammaglobulinemia was linked to these autoimmune conditions.

## 8. INTERVENTION AND FOLLOW-UP

The patient was reviewed by a rheumatologist and started on azathioprine 100 mg daily and hydroxychloroquine 200 mg daily. After three months, significant improvement in inflammatory markers was observed after three months of treatment with hydroxychloroquine and azathioprine, consistent with the typical response timeline of 8–12 weeks for these medications in autoimmune diseases: ESR reduced to 20 mm/hr, CRP < 2 mg/l, and white cell counts normalized to 4.5 × 10^9/L. The renal function remained stable.

A cardiologist also evaluated her and diagnosed rheumatic mitral heart disease, which was complicated by heart failure. She was started on bisoprolol 1.25 mg daily, valsartan 40 mg daily, and furosemide. Although she initially declined mitral valve replacement surgery, further discussion with her family physician led to her agreement for the procedure, considering the risk of worsening pulmonary hypertension if left untreated.

Due to chronic joint pain, inflammatory conditions, and heart failure symptoms, the patient developed anxiety and depression and was prescribed escitalopram 10 mg daily with psychological support. Her mood improved significantly during follow-up appointments.

## 9. DISCUSSION

This case highlights the critical importance of a systematic and multidisciplinary approach in distinguishing autoimmune diseases from hematologic malignancies in patients presenting with hypergammaglobulinemia and pancytopenia. Initial findings, including elevated inflammatory markers, polyclonal hypergammaglobulinemia, and cytopenias, raised strong suspicion for multiple myeloma, which remains a common diagnosis in such scenarios due to its association with monoclonal gammopathy. However, the absence of a monoclonal band on serum protein electrophoresis and a normal kappa/lambda ratio shifted the focus toward an autoimmune etiology, ultimately confirmed by autoantibody profiles consistent with SLE and SS.

Polyclonal hypergammaglobulinemia arises from chronic immune activation, which is a feature of autoimmune diseases such as SLE and SS, as well as chronic infections and liver diseases. In contrast, monoclonal gammopathies, such as multiple myeloma or MGUS, exhibit a monoclonal protein spike on electrophoresis, reflecting clonal plasma cell proliferation.^15^

The coexistence of SLE and SS is not uncommon, with studies estimating overlap in 9%–33% of patients with SLE.^12^ Shared pathophysiological mechanisms, including B-cell hyperactivity, autoantibody production, and lymphocytic infiltration, contribute to overlapping features that complicate diagnosis and management. Polyclonal hypergammaglobulinemia in these conditions reflects systemic immune dysregulation and chronic inflammation, which are frequently accompanied by significant clinical manifestations such as pancytopenia, arthritis, and mucosal dryness, as observed in this case. In addition, patients with SLE are at a modestly increased risk of developing hematologic malignancies, including multiple myeloma, necessitating ongoing vigilance.^18^

Genetic testing in this patient was essential to exclude mutations associated with hematologic malignancies, such as JAK2 or CALR mutations, which could explain myeloproliferative neoplasms or other findings. Although no pathogenic mutations were identified, the inclusion of genetic studies highlights the importance of thorough malignancy exclusion in complex cases. Emerging evidence also suggests a genetic predisposition to autoimmune diseases, including HLA-DR and other loci, which may explain overlapping autoimmune conditions such as SLE and SS.^19^ The patient showed significant improvement within three months of initiating immunosuppressive therapy, which is consistent with expected treatment response timelines for autoimmune conditions. Hydroxychloroquine, a cornerstone in the management of SLE, and azathioprine, an immunosuppressant effective in overlapping autoimmune diseases, likely contributed to this improvement. While early biochemical responses are common within three to six months, sustained clinical remission often requires long-term management and regular monitoring for complications.

## 10. LIMITATIONS

This case report has several limitations specific to this patient:

1. **Lack of Long-Term Follow-Up:** Although the patient showed significant improvement within three months of treatment initiation, the absence of long-term follow-up data limits the ability to assess sustained disease control and potential complications, such as progression to lymphoma or other malignancies associated with SS. However, comprehensive investigations were conducted to avoid missing any differentials.
2. **Missing Serial Laboratory Data:** Although initial laboratory results were comprehensive, serial measurements of polyclonal hypergammaglobulinemia and autoantibody titers were not available. These could have provided additional insights into the patient’s disease trajectory and treatment response.
3. **Limited Imaging Correlation:** Imaging findings, such as hypermetabolic activity in cervical lymph nodes and the L4 vertebra, were attributed to reactive lymphoid hyperplasia. However, additional confirmatory studies, such as a biopsy of metabolically active lymph nodes, may have strengthened the diagnostic workup.
4. **No Genetic Studies for Autoimmune Predisposition:** Although genetic testing excluded hematologic malignancy-related mutations (e.g., JAK2, CALR), no studies were performed to investigate potential genetic predispositions to autoimmune diseases, which may have contributed to the patient’s overlapping SLE and SS.
5. **Single Case Report:** Being a single case report, the findings are not generalizable to a broader population. Further studies with larger sample sizes are needed to confirm these observations.
6. **Retrospective Nature:** Retrospective case studies can be subject to information bias and may not capture all relevant clinical details.
7. **Causation:** Observational reports, such as this one, cannot establish causation but can only suggest associations and highlight diagnostic challenges.

## 11. CONCLUSION

Patients presenting with high total protein and low albumin levels due to elevated globulins should undergo protein electrophoresis to differentiate between polyclonal and monoclonal hypergammaglobulinemia. Polyclonal hypergammaglobulinemia, when accompanied by elevated inflammatory markers, should prompt consideration of autoimmune diseases such as SLE and SS. Early autoimmune screening is crucial in avoiding misdiagnosis and ensuring timely and appropriate management.

The coexistence of autoimmune diseases, as seen in this case, requires vigilant monitoring and a multidisciplinary approach to prevent complications and improve patient outcomes. While this case underscores the importance of thorough diagnostic evaluation, it also highlights the need for long-term follow-up and further studies to elucidate the interplay between autoimmune disorders and hypergammaglobulinemia.

## Figures and Tables

**Table 1.** Basic screening lab results.

**Parameter**	**Result**	**Reference range**	
Hb	9.3 g/dL	12.5-18.0 g/dL	Low
WBC	3.3 × 10^9/L	4-11 × 10^9/L	Low, leukopenia
MCV	69 fl	80-100 fl	Low, microcytosis
MCH	20 pg/cell	27-33 pg	Low, hypochromia
Platelet	122,000 K/uL	150-400 K/uL	Low, thrombocytopenia
ESR	105 mm/hr	<30 mm/hr	High
CRP	137.7 mg/L	0-5 mg/L	High,
INR	1.1	0.8–1.2	Normal
APTT	38 s	21-35 s	Slightly elevated, borderline prolonged
Serum corrected Ca	2.78 mmol/L	2.2-2.78 mmol/L	Upper normal
Serum total protein	107 g/L	60-80 g/L	High, hyperproteinemia
Serum Albumin	32 g/L	35-50 g/L	Low, hypoalbuminemia
HbA1c	5.10%	4.5-5.7%	Normal
HB electrophoresis	Normal Hb pattern	Normal Hb pattern	Normal
Urea	3.5 mmol/L	2.5-7.8 mmol/L	Normal
Cr	60 µmol/L	44-80 µmol/L	Normal
Na	134 mmol/L	133-146 mmol/L	Normal
K	4.4 mmol/L	3.5-5.3 mmol/L	Normal
Chloride	101 mmol/L	95-108 mmol/L	Normal
Bicarbonate	26 mmol/L	22-29 mmol/L	Normal
Phosphorus	0.98 mmol/L	0.8-1.5 mmol/L	Normal
Mg	0.81 mmol/L	0.7-1 mmol/L	Normal
Uric Acid	344 µmol/L	300-400 µmol/L	Normal
Alkaline phosphatase	87 u/L	35-104 u/L	Normal
ALT	25 u/L	0-33.0 u/L	Normal
AST	19 u/L	0-32.0 u/L	Normal
LDH	151 IU/L	105-333 IU/L	Normal

**Table 2.** Autoantibodies screening results.

**Parameter**	**Result**	**Reference range**	**Interpretation**
RH	H 39	< 15 IU/mL	High
Anti CCP	>8	< 20 u/mL	Negative
Anti Nuclear Ab (ANA)	Positive	Negative	Positive
ANA Titer	≥1:1280	1:40–1:160	Elevated
ANA Pattern	Speckled	-	Speckled Pattern
Anti dsDNA Ab	1600 IU/mL	< 30 IU/mL	Positive
ANA CTD Int	Positive	Negative	Positive
CENP	6.4	< 7 units/mL	Negative
CENP Int	Negative	Negative	Negative
Scl-70	0.6	< 7 units/mL	Negative
Scl-70 Int	Negative	Negative	Negative
Jo-1	0.8	< 7 units/mL	Negative
Jo-1 Int	Negative	Negative	Negative
LA (Lupus Anticoagulant)	>320.0	< 7 units/mL	Positive
LA Int	Positive	Negative	Positive
Rib-p	2.7	< 7 units/mL	Negative
Rib-p Int	Negative	Negative	Negative
RNP	1.7	< 7 units/mL	Negative
RNP Int	Negative	Negative	Negative
RO (SS-A)	>240.0	< 7 units/mL	Positive
RO Int	Positive	Negative	Positive
SMD (Smith)	2.5	< 7 units/mL	Negative
SMD Int	Negative	Negative	Negative

**Table 3.** General immunology results.

**Parameter**	**Result**	**Reference range**	**Interpretation**
IgG	H 53.06	6.0-16.0 g/L	High
IgA	H 6.90	0.8-3.0 g/L	High
IgM	0.48	0.4-2.5 g/L	Normal
C3	1.37	0.75-1.75 g/L	Normal
C4	H 0.48	0.15-0.45 g/L	Slightly elevated

**Table 4.** Imaging and biopsy results.

**Imaging/procedure**	**Results**
ECG	Normal sinus rhythm, P mitrale indicating LAH
Echocardiography	Increased pulmonary artery pressure, LVEF 54%
CT (Neck, Chest, Abdomen, Pelvis)	Right thyroid nodule, mild pelvic congestion, ground glass opacities in right lung
NM Whole Body FDG PET-CT	Hypermetabolic activity in the L4 vertebra, anterior cervical lymph nodes, and left adrenal uptake; no high-grade malignancy
Bone marrow aspirate and biopsy	Normal hybridization pattern, trilineage hematopoiesis, mild fibrosis, multiple lymphoid aggregates
Right Submandible FNA	Benign/reactive lymph node, mixed lymphocytes, scanty macrophages
